# Supplementary material for: A transcriptional atlas of early Arabidopsis seed development suggests mechanisms for inter-tissue coordination
Source: Nat Plants. 2026 May 21;12(5):1133–49. doi: 10.1038/s41477-026-02295-8 (PMC13197225; doi:10.1038/s41477-026-02295-8)
Supplement: Supplementary file 2 — Reporting Summary [file 41477_2026_2295_MOESM2_ESM.pdf]

Corresponding author(s): Mary Gehring
Last updated by author(s): Feb 12, 2026

Reporting Summary

Nature Portfolio wishes to improve the reproducibility of the work that we publish. This form provides structure for consistency and transparency in reporting. For further information on Nature Portfolio policies, see our Editorial Policies and the Editorial Policy Checklist.

Statistics

For all statistical analyses, confirm that the following items are present in the figure legend, table legend, main text, or Methods section.

n/a	Confirmed	
☐	☒	The exact sample size (*n*) for each experimental group/condition, given as a discrete number and unit of measurement
☒	☐	A statement on whether measurements were taken from distinct samples or whether the same sample was measured repeatedly
☐	☒	The statistical test(s) used AND whether they are one- or two-sided<br>*Only common tests should be described solely by name; describe more complex techniques in the Methods section.*
☒	☐	A description of all covariates tested
☐	☒	A description of any assumptions or corrections, such as tests of normality and adjustment for multiple comparisons
☐	☒	A full description of the statistical parameters including central tendency (e.g. means) or other basic estimates (e.g. regression coefficient) AND variation (e.g. standard deviation) or associated estimates of uncertainty (e.g. confidence intervals)
☐	☒	For null hypothesis testing, the test statistic (e.g. *F*, *t*, *r*) with confidence intervals, effect sizes, degrees of freedom and *P* value noted<br>*Give P values as exact values whenever suitable.*
☐	☒	For Bayesian analysis, information on the choice of priors and Markov chain Monte Carlo settings
☒	☐	For hierarchical and complex designs, identification of the appropriate level for tests and full reporting of outcomes
☐	☒	Estimates of effect sizes (e.g. Cohen's *d*, Pearson's *r*), indicating how they were calculated

Our web collection on statistics for biologists contains articles on many of the points above.

Software and code

Policy information about availability of computer code

Data collection	BD FACS DIVA 8.0.1<br>ZEN 2012 SP5 FP3 (black)
Data analysis	ImageJ version 2.16.0<br>CellRanger 7.1.0<br>STAR 2.7.1a<br>OrthoFinder 2.5.4<br>PAML 4.9<br>R version 4.2.1<br>Scripts for analyses are deposited in GitHub at https://github.com/Gehring-Lab/seed_atlas_2025, which implement the following R packages:<br>biomartr_1.0.7<br>data.table_1.14.10<br>seqinr_4.2-30<br>clusterProfiler_4.7.1.002<br>pheatmap_1.0.12<br>patchwork_1.2.0<br>monocle3_1.3.7<br>org.At.tair.db_3.16.0<br>limma_3.54<br>cluster_2.1.6<br>ggraph_2.1.0

scran_1.26.2
lubridate_1.9.3
purrr_1.0.2
tidyr_1.3.0
tidyverse_2.0.0
Rcpp_1.0.11
optparse_1.7.3
scCustomize_2.0.1
SoupX_1.6.2
scDblFinder_1.12.0
SummarizedExperiment_1.28.0
GenomicRanges_1.50.2
IRanges_2.32.0
BiocGenerics_0.44.0
matrixStats_1.1.0
SeuratObject_5.0.1
orthologr_0.4.2
RColorBrewer_1.1-3
enrichplot_1.18.4
magrittr_2.0.3
SeuratWrappers_0.4.0
cowplot_1.1.2
AnnotationDbi_1.60.2
clustree_0.5.1
bluster_1.8.0
scuttle_1.8.4
forcats_1.0.0
readr_2.1.4
tibble_3.2.1
sp_2.1-2
Seurat_5.0.0
MatrixGenerics_1.10.0
S4Vectors_0.36.2
GenomeInfoDb_1.37.1
Biobase_2.58.0
SingleCellExperiment_1.20.1
dplyr_1.1.4
ggplot2_3.4.4
argparse_2.2.2
stringr_1.5.1
harmony_1.2.0
tibble_3.2.1

For manuscripts utilizing custom algorithms or software that are central to the research but not yet described in published literature, software must be made available to editors and reviewers. We strongly encourage code deposition in a community repository (e.g. GitHub). See the Nature Portfolio guidelines for submitting code & software for further information.

Data

Policy information about availability of data

All manuscripts must include a data availability statement. This statement should provide the following information, where applicable:

- Accession codes, unique identifiers, or web links for publicly available datasets
- A description of any restrictions on data availability
- For clinical datasets or third party data, please ensure that the statement adheres to our policy

All sequencing data is available in NCBI GEO GSE295007. Scripts for analyses are deposited in GitHub at https://github.com/Gehring-Lab/seed_atlas_2025. A browser for the data is available at https://seedatlas.wi.mit.edu/

Research involving human participants, their data, or biological material

Policy information about studies with human participants or human data. See also policy information about sex, gender (identity/presentation), and sexual orientation and race, ethnicity and racism.

Reporting on sex and gender

Use the terms sex (biological attribute) and gender (shaped by social and cultural circumstances) carefully in order to avoid confusing both terms. Indicate if findings apply to only one sex or gender; describe whether sex and gender were considered in study design; whether sex and/or gender was determined based on self-reporting or assigned and methods used.
Provide in the source data disaggregated sex and gender data, where this information has been collected, and if consent has been obtained for sharing of individual-level data; provide overall numbers in this Reporting Summary. Please state if this information has not been collected.
Report sex- and gender-based analyses where performed, justify reasons for lack of sex- and gender-based analysis.

Reporting on race, ethnicity, or

Please specify the socially constructed or socially relevant categorization variable(s) used in your manuscript and explain why

Reporting on race, ethnicity, or other socially relevant groupings	*they were used. Please note that such variables should not be used as proxies for other socially constructed/relevant variables (for example, race or ethnicity should not be used as a proxy for socioeconomic status).*<br>*Provide clear definitions of the relevant terms used, how they were provided (by the participants/respondents, the researchers, or third parties), and the method(s) used to classify people into the different categories (e.g. self-report, census or administrative data, social media data, etc.)*<br>*Please provide details about how you controlled for confounding variables in your analyses.*
Population characteristics	*Describe the covariate-relevant population characteristics of the human research participants (e.g. age, genotypic information, past and current diagnosis and treatment categories). If you filled out the behavioural & social sciences study design questions and have nothing to add here, write "See above."*
Recruitment	*Describe how participants were recruited. Outline any potential self-selection bias or other biases that may be present and how these are likely to impact results.*
Ethics oversight	*Identify the organization(s) that approved the study protocol.*

Note that full information on the approval of the study protocol must also be provided in the manuscript.

Field-specific reporting

Please select the one below that is the best fit for your research. If you are not sure, read the appropriate sections before making your selection.

☒ Life sciences ☐ Behavioural & social sciences ☐ Ecological, evolutionary & environmental sciences

For a reference copy of the document with all sections, see nature.com/documents/nr-reporting-summary-flat.pdf

Life sciences study design

All studies must disclose on these points even when the disclosure is negative.

Sample size	We did not select a sample size for snRNA-sequencing data collection. We assessed atlas cell type coverage by the detection of known, rare cell types in the embryo. Each snRNA-sequencing library contains 8-11k nuclei extracted from >500 seeds, and there are two libraries for each timepoint.
Data exclusions	We excluded genes and nuclei that did not meet our abundance or quality thresholds, respectively. Genes detected in less than 10 nuclei were removed, and snRNA-seq profiles with less than 250 genes filtered out. Following the recommendation from Heumos et al. 2023, we further identified and removed “outlier” nuclei as those with a gene/nucleus or a transcript/nucleus that differs by 5 median absolute deviations from the rest of the sample. We also removed nuclei that were predicted doublets by DoubletFinder. Nuclei found in low-quality clusters by low gene/nucleus metrics were also removed.
Replication	We generated two biological replicates for each timepoint, and all replicates show high transcriptional correlation.<br>We identified cluster-specific markers using differential expression analysis and used HCR-RNA FISH to corroborate our cluster annotations with an orthogonal method.
Randomization	This is not relevant to our study because we did not perform experiments on any treatment/control groups.
Blinding	This is not relevant to our study because we did not perform experiments on any treatment/control groups.

Reporting for specific materials, systems and methods

We require information from authors about some types of materials, experimental systems and methods used in many studies. Here, indicate whether each material, system or method listed is relevant to your study. If you are not sure if a list item applies to your research, read the appropriate section before selecting a response.

Materials & experimental systems

n/a	Involved in the study	
☒	☐	Antibodies
☒	☐	Eukaryotic cell lines
☒	☐	Palaeontology and archaeology
☒	☐	Animals and other organisms
☒	☐	Clinical data
☒	☐	Dual use research of concern
☐	☒	Plants

Methods

n/a	Involved in the study	
☒	☐	ChIP-seq
☐	☒	Flow cytometry
☒	☐	MRI-based neuroimaging

Plants

Seed stocks: Arabidopsis thaliana Col-0

Novel plant genotypes: No new plant genotypes were generated.

Authentication: No authentication methods were used.

Flow Cytometry

Plots

Confirm that:

☒ The axis labels state the marker and fluorochrome used (e.g. CD4-FITC).

☒ The axis scales are clearly visible. Include numbers along axes only for bottom left plot of group (a 'group' is an analysis of identical markers).

☒ All plots are contour plots with outliers or pseudocolor plots.

☒ A numerical value for number of cells or percentage (with statistics) is provided.

Methodology

Sample preparation: Two biological replicates, (different plants hand-pollinated and processed for snRNA-seq on different days) each containing seeds isolated from 10-15 siliques (500-800 seeds), were collected for each timepoint, producing six replicates total. Seeds were dissected into 150 uL cold extraction buffer on ice (1x Partec CyStain UV Precise P nuclei extraction buffer (Sysmex #05-5002), 4% BSA, 1mM DTT, 1:100 protease inhibitor cocktail for plants (Sigma #P9599), and 1:30 Protector RNase inhibitor (Fisher Scientific #NC1877809)). Seeds were mechanically dissociated in 1.5 mL tubes by grinding with an Axygen pestle (Corning #PES-15-B-SI) for 10 turns. The nuclei suspension was filtered through a 30 um cell strainer (Fisher Scientific # NC9682496), prewet with Partec CyStain UV Precise P staining buffer (Sysmex #05-5002), into a 5 mL tube for fluorescence activated nuclei sorting (FANS). The strainer was rinsed with Partec CyStain UV Precise P staining buffer into the 5 mL tube to bring the final suspension volume to 1 mL. 1 uL of 1mg/mL DAPI (ThermoFisher Scientific #62248) was added to increase nuclear signal. Nuclei were purified and concentrated by FANS on a BD FACS ARIA Cell Sorter using a 70 um nozzle chip. We gated on 2C, 3C, 4C, 6C, 8C, and 16C peaks (Supplementary Fig. 9). Nuclei were sorted into 30-50 uL collection buffer (PBS-4% BSA) in a 1.5 mL tube and concentration was assessed using the ARIA nuclei count and by manual counting on a Neubauer Improved hemocytometer.

Instrument: BD FACSAria II flow cytometer

Software: BD FACS DIVA 8.0.1

Cell population abundance: We collected 2C, 4C, 3C, 6C, 8C, and 16C peaks. The collected population was 48%, 35%, and 32% of total events at 3, 5, and 7, days after pollination, respectively. Final proportions of sorted nuclei types, after snRNA-sequencing analysis, are: 11% embryo, 24% endosperm, 63% seed coat, and 2% unfertilized ovule and funiculus in the combined atlas dataset.

Gating strategy: The nuclei suspension was first gated based on size (FSC-A) and DNA content (DAPI-A). We collected all nuclei in diploid peaks, which are seed coat and embryo-derived (2C, 4C), triploid peaks, which are endosperm-derived (3C, 6C), and endoreduplicated/potential nuclei fusion peaks (8C, 16C). See supplementary Figure 5 for our gating strategy.

☒ Tick this box to confirm that a figure exemplifying the gating strategy is provided in the Supplementary Information.
